# The Dictionary of Foods and Drinks

**Published:** 1855-09

**Authors:** 


### THE DICTIONARY OF FOODS AND DRINKS;

### A COMPENDIUM OF ALIMENTARY SUBSTANCES IN USE THROUGHOUT THE WORLD: THEIR HISTORIES, DIETETIC PROPERTIES, AND ADULTERATIONS.

### PART II.

ADULTERANTS, in regard to food, is a term used to express agents employed for the purpose of modifying or deteriorating any alimentary substance. Adulterants are derived from the animal, vegetable, and mineral kingdoms. The uses to which they are applied are various. Sometimes they are used to increase quantity, the adulterant being much cheaper than the article of food with which it is mixed. An example of this kind is given in the adulteration of coffee with roasted corn or acorns. In other cases the adulterant is intended to supersede some other more expensive substance for imparting a flavour; in this way gentian is used instead of the hop for giving a bitter flavour to beer. In a third class of cases, the adulterating agent is resorted to as means of modifying colours, and thus sulphate of copper is used for giving a green appearance to common pickles. In other instances, the adulterant is accidentally mixed with the true article, and an organic adulterant may form accidentally, as is the case with the acarus or sugar mite, which is developed in moist brown sugars. As regards the effects of adulterants on the body, it is to be observed, that they are not all necessarily hurtful. Some are simple, and are themselves common articles of food. Others are not at once injurious, but become so from long continued use; while a third class, including those of the mineral kingdom specially, are highly deleterious and even poisonous. It does not seem, indeed, that those who practise adulteration consider the question of the effects of the adulterant on health. Upwards of two hundred substances admit of being classed as adulterants, of which the following are the principal, arranged in alphabetical order, and considered in regard to the objects for which they are used and their effects on the economy.

ACARUS, the mite, an animal of the order of *arachnida,* the mite. There are several varieties: as the sugar mite, meal mite, and cheese mite. It is doubtful whether they exert any injurious influence on the body, but Dr. Hassall has raised the question whether the mite which infests coarse sugars may not be the cause of grocers' itch. The presence of the mite in meal indicates that the flour is not fit for consumption. See *Acarus,* part I.

ACONITE *(Aconitum napellus),* a plant of the natural order *Ranunculaceæ,* used in Belgium, France, and Switzerland, according to Remer, to colour sweetmeats. The plant is a virulent poison, diminishing the force of the circulation, weakening the muscular system, and destroying common sensation. The root has sometimes been used in mistake for horse-radish, and has caused death.

ACORN, fruit of the oak (*Quercus pedunculata,* natural order *Cupuliferæ*). Used in the adulteration of coffee and chicory. It is not injurious to health, and was formerly thought to have nutritious properties. See *Acorn,* part I.

ALBUMEN, an alimentary substance existing in organised structures. There are two kinds of albumen, animal and vegetable. The best specimen of animal albumen is the white of egg; vegetable albumen is the chief agent in adulteration. It is not an intentional adulterant, but occurs in most brown sugars naturally. The effects of the albumen itself are not injurious, since it is simply nutritious in its character. Mixed with the sugar, however, it lessens the special sweetening properties of that substance, and renders it liable to undergo fermentation and putrefaction.

ALCOHOL (spirit of wine) is added to cider and other stimulating beverages to give strength, and render them more intoxicating.

ALKANET (*Anchusa tinctoria*), a plant of the natural order *Boraginaceæ,* used to colour butter. Though not injurious, it deceives as to the quality of the butter, when this is largely adulterated with lard, tallow, or other animal fats.

ALLSPICE, the powdered fruit of the *Eugenia Pimenta,* a plant of the natural order *Caryophyllaceæ;* it is rolled up with rye, starch, mustard and rape-seed to form spurious pepper-corns, in France, under the name of Lyons pepper.

ALOE. The leaves of some of the genus *aloe,* natural order *Liliaceæ,* natives of southern Africa, are dried, broken into small pieces, and, being mixed with a paste of gum and starch, are reduced into a coarse powder, and used in the adulteration of tea. The aloe leaf gives a flavour somewhat resembling tea. The effect of this adulteration is not well known. Some of the aloe plants yield the purgative extract known as aloes: it is doubtful, however, whether the dried leaves contain any purgative or other deleterious principle.

ALUM, (*Sulphate of Alumina and Potass,*) a crystalline salt found naturally in Italy in alum shale. Alum is constantly used in the adulteration of bread or flour, for the purpose of imparting whiteness and favouring the introduction and retention of water. In bread it is mixed in the form of "hards" or "stuff", a mixture merely of alum itself and salt. It is used also in the adulteration of English keg lard, but is rarely found in the Irish keg lard. In the Louvain alum is employed to render clear and give head to beer, and to fine gin. The effects of alum on the animal body are of a styptic or astringent kind; hence it is sometimes usefully applied as a lotion to stop bleeding from wounds, and is also given internally to check intestinal fluxes. As, however, it forms no part of the body, and has these styptic effects, it is an injurious as well as a fraudulent adulterant. Its continual use gives rise to constipation and to disordered and enfeebled digestion.

ANTIMONY, a metallic element, found by M. Morin in the famous Strasbourg *pâtés de foies gras.* It is derived from the custom of adding sulphuret of antimony to the food of the geese, to make them become fat more quickly. Chevallier does not think the quantity conveyed in the food sufficient to be hurtful.

ANTWERP BLUE, a colouring matter (resembling Prussian blue) used to colour sugar confectionery; it is not deleterious except when taken for a long time.

APPLES, fruit of the common apple tree, used largely in the adulteration of marmalade; the adulteration is simply fraudulent.

ARMENIAN BOLE, a clay coloured by iron, used extensively in the adulteration of anchovies, potted beef, potted bloaters, anchovy paste, and tomato sauce. It has been found in anchovies to such an extent as to be obtainable by spoonfuls. The object is to give colour. In small quantities, Armenian bole is not injurious. At one time it had a great reputation as a medicine, being considered a tonic and astringent, and a medicine which expelled poisons. It possibly may possess some of the properties of a ferruginous tonic, and its effects are not deleterious unless taken for a considerable period, when the digestive organs would suffer from its use.

ARNOTTO, the dried pulp of the seeds of the *Bixa Orellana,* a small tree common in the West India islands. It is used to give a yellow colour to cheese and butter. It does no harm to the body, but may deceive as to the quality of the article sold. Cheshire cheese and Leicester fair cheese are mostly deeply tinged with this very unnecessary substance. It is proper to observe, that what is sold in the shops as arnotto often contains no trace of that substance, being a compound of turmeric, or some other colouring matter, as red lead mixed up with soap, or with potash and a fish oil.

ARROW ROOT, the powdered root of the *Maranta arundinacea,* natural order *Marantaceæ,* a South African plant. It is used in the adulteration of chocolate, cocoa, cinnamon, sugar, and some confections. The adulteration is a fraud, but the effects are not injurious to health, the adulterant being in itself a nutritive article of diet.

ARSENIC. The compounds of this metal are found in various cases as adulterants, being sometimes present as the result of accident, in other cases being intentionally introduced. The common white arsenic (*arsenious acid*) may be accidentally present in unfermented bread, being derived from the use of impure muriatic or hydrochloric acid. According to Chevallier, cheese is sometimes washed over with a solution of arsenic, to preserve it from insects. He relates that, in 1840 or 1841, several cheesemongers and inhabitants of Châtillon in France were attacked with severe vomiting and pains in the bowels after eating cheese prepared in this manner. A similar event occurred in Paris in 1854. Wheat and other grain are sometimes steeped in a solution of arsenic to preserve them from insects when sown; the grain thus prepared has been stated to be poisonous to birds, and would also be so to man, if eaten by accident. The *red sulphuret of arsenic* (realgar), and the *yellow sulphuret* (king's yellow), are used to colour confectionery; but, on account of their poisonous properties, the use of these compounds for that purpose is prohibited in France. The country people in Upper Styria are said to use white arsenic as a condiment, to the extent of as much as two grains daily. The alleged effect is to promote the appetite and general vigour. One grain of arsenious acid leads to serious effects; and four grains have been known to destroy life. In small and continued doses, arsenic may be taken for a considerable time with moderate impunity, but it is liable to produce deleterious results. It is, therefore, one of the most dangerous of adulterants.

ASHES OF WOOD, according to Chevallier, are sometimes added to cider, probably with the object of neutralising acidity; an effect which is dependent on the alkali which they contain.

ASPARAGUS, a plant of the natural order *Liliaceæ*. The berries are used to colour butter. Though not injurious, their use deceives as to the quality of the butter, especially when this is largely adulterated with animal fats. The young shoots of asparagus are diuretic, and communicate a peculiar odour to the urine.

BARLEY, the grain of the *Hordeum distichon*, natural order *Graminaceæ*. The flour is sometimes mixed with wheat flour and oatmeal; it is used also to adulterate mustard. It is introduced into watered milk to increase its density, give a creamy appearance, or remove the bluish tint. It is detected by means of the microscope: the small starch granules of barley being three or four times smaller than the corresponding grains of wheat starch, and many of the large grains presenting concentric rings, which are absent in the granules of wheat starch.

BASTARD PLANE, Leaves of, are dried, broken into small pieces, and usually mixed with a paste of gum and starch, then reduced to powder, and used in adulterating tea.

BEANS, common plants of the genus *Faba*, natural order *Leguminosæ*. The flour is used in the adulteration of wheat-flour, sago, and, when burnt, in the adulteration of coffee. It may be detected in flour by the bean odour evolved when a little boiling water is poured on the flour to be examined. Common horse-beans, in powder, are used to adulterate mustard. Haricot beans, rape or linseed, grey fecula, and potato blossoms are powdered and mixed in France to form spurious pepper. Haricot beans, roasted, are used to adulterate honey. Beans are considered to have highly nutritious qualities. These adulterations are simply fraudulent.

BEECH (*Fagus sylvatica*), a tree of the natural order *Amentaceæ*. The leaves are dried, broken into small pieces, and usually mixed with a paste of gum and starch, then reduced to powder, and used in adulterating tea. The oil of the nuts (beech-nut oil) is used as adulterant of, or substitute for, olive oil. The powder of beech-nuts and hempseed, bran, and husks of pepper, are used in France to form a spurious pepper. These adulterations are simply fraudulent.

BEER, Sour, used in adulterating vinegar.

BEET (*Beta vulgaris*), a plant of the natural order *Chenopodiaceæ.* The juice of the red beet is used to colour gooseberry jelly. Beet-root is also formed into rolls, and roasted, to form spurious roasted onions, in France. Large quantities of sugar are made from beet on the continent. Its use as an adulterant is simply fraudulent.

BISCUIT POWDER, Burnt, used in adulteration of chicory, coffee, and cocoa. Burnt biscuits or crusts are substituted for cocoa nibs. The adulteration is simply fraudulent.

BLOOD of animals is used, but less frequently than formerly, in the manufacture of loaf-sugar. Its presence does no harm. Blood has been recommended as a medicine, on account of the iron it contains.

BONES, ground, said to be used in the adulteration of flour. The practice is principally fraudulent: the matter introduced is principally phosphate and carbonate of lime. See *Lime*.

BOX (*Buxus sempervirens*), a tree of the natural order *Euphorbiaceæ.* The leaves and bark are used for bittering and colouring beer. They are not known to have poisonous effects.

BRAINS of Animals, said to be used in the adulteration of milk. This, however, is denied by Normandy.

BRAN, used to adulterate flour and linseed meal. It is supposed to have an aperient effect.

BREAD CRUMBS, used to give a marbled appearance to cheese.

BRICK DUST, used in the adulteration of cocoa, chocolate, chicory, and Cayenne pepper. It acts as a mechanical irritant on the alimentary canal.

BROMINE, a non-metallic elementary substance. Compounds of bromine are sometimes accidentally present in common salt, but not in quantities sufficient to produce any deleterious effect.

BRONZE, a compound of copper and tin. The powder is used to colour sweetmeats. It is highly deleterious.

BRUNSWICK GREEN (an *Oxichloride of Copper*), a colouring matter used to colour sugar confectionery. See *Copper*.

BUCK-BEAN (*Menyanthes trifoliata*), a plant of the natural order *Gentianaceæ*, is used for bittering and colouring beer in some parts of Germany, particularly in Silesia and the adjacent provinces. It has been considered a stomachic; it is not deleterious.

CARAMEL, or Burnt Sugar, is used to colour brandy. Its use is not deleterious.

CARROT, (*Daucus carota*,) a plant of the Natural Order *Um-*

*belliferæ*. The root is used in adulterating chicory and coffee. The juice is used to colour butter. Carrots are formed into rolls, and roasted to form spurious roasted onions in France. Boiled carrots are introduced into milk to increase its density, give a creamy appearance, or remove the bluish tint.

CASSIA, the bark of the *Cinnamomum cassia*, a tree of the natural order *Lauraceæ*, is substituted for cinnamon, which it resembles in properties. The oil of cassia is used to adulterate gin. It is not deleterious.

CATECHU or Japan earth, an extract prepared from the wood of the *Acacia catechu*, a tree of the natural order *Leguminosæ*, common in various parts of the East Indies and Jamaica. It is sometimes mixed with exhausted tea leaves, to impart astringency and colour to the infusion. It formed the principal ingredient, according to Dr. Hassall, of "La Veno Beno", an article sold some years ago as a substitute for tea. Catechu is an astringent: its continued use is likely to injure the digestive organs.

CAYENNE PEPPER, the powdered fruit of the *Capsicum frutescens*, a plant of the natural order *Solanaceæ*. It is used in adulterating ginger, gin, and rum. It is liable to act as an irritant.

CHALK (*Carbonate of Lime*). See *Lime*.

CHARCOAL of wood has been found with earthy matter, to the amount of 15 per cent., in chicory, by Dr. Normandy.

CHEESE, ROTTEN, treated with sulphuric acid and bichromate of potash, has been used to produce a very fragrant fruity essence, which is substituted for the essence obtained from the pine apple.

CHESNUT, common horse chesnut (*Æsculus hippocastanum*), a tree of the natural order *Æsculaceæ*. The leaves are dried, broken into small pieces, and mixed with a paste of gum and starch, then reduced to powder, and used in adulterating tea. The adulteration is simply fraudulent.

CHICORY, a perennial plant, the *Cichorium intubus*, natural order *Compositæ*. The root is used largely in adulterating coffee, in proportions varying from one-third or less to entire substitution. It is detected by the microscope; and, if in large quantities, by its forming a pellet when rolled between the moistened fingers: coffee will not knead. Chicory is also detected by placing the suspected article on water; chicory sinks and colours the water; coffee floats and gives no colour, owing to its being surrounded by oil. It is also used in the roasted form for bittering and colouring beer. Chicory is supposed by some to be nutritious. It is more irritant than coffee, and, when its use is long continued, it excites indigestion.

CHINA CLAY or Kaolin (a *Silicate of Alumina*), is said to be used in the adulteration of tea by the Chinese. Mr. Warrington has detected a white powder in some samples of tea, which, from analysis, he concludes to have been in part composed of this substance. Its effects would be the same as those of alum.

CHROME YELLOW (*Chromate of Potash*). See *Potash.*

CIDER has been used in adulterating vinegar.

CINNABAR, or Vermillion (*Sulphuret of Mercury*), used to adulterate chocolate and cayenne pepper. See *Mercury.*

CLAY (*Silicate of Alumina*) has been found in bread by Dr. Normandy. It is also used to adulterate linseed meal and salt. Ground clay has also been used to adulterate pepper.

CLOVE (*Caryophyllus aromaticus*), a tree of the natural order *Myrtaceæ.* The broken stalks or peduncles, reduced to powder, are sometimes mixed with the powder of the unexpanded flower, the part in legitimate use. Cloves are used to give colour and bitterness to beer. The clove is an aromatic: its use is not deleterious.

COBALT, a metallic substance. The salts of this metal have been used to give a blue colour to confectionery. Being poisonous, its use for this purpose has been prohibited in France.

COCCULUS INDICUS, or *Anamirta Cocculus* (sometimes called Levant nut) a plant of the natural order *Menispermaceæ.* The solution of the extract is used to adulterate stout and porter, rum, and gin, to produce intoxicating effects. It contains about 2 per cent. of picrotoxin, and is used by poachers to destroy pheasants and fishes. Its presence in rum has caused death. It is also stated to be present in ales.

COCHINEAL (*Coccus cacti*), an insect of the hemipterous order. The dried insect is used in colouring tomato sauce and sugar confectionery. It possesses no deleterious properties.

COFFINA, a powder supposed to be made from cassia seeds, recommended to be used with chicory in place of coffee.

COPPER, a well known metal. From the readiness with which it is acted on by acids, some articles of food, as preserves, beer, milk, butter, or salt, made or kept in vessels made of this metal, become impregnated with its salts. *Metallic copper*, in powder, and *carbonate of copper*, have been used to colour sugar confectionery; their use is prohibited in France. Pickles are also boiled sometimes with their vinegar in copper vessels; by this process an acetate of copper (verdigris) is formed, which gives a greenness to the pickles; bottled fruits and vegetables are also boiled in copper for the same purpose. Copper is sometimes present, to a poisonous extent, in oysters and other shell-fish, being derived from the bottoms of vessels. It may be accidentally present in flour, being derived from any part of the mill which may contain it. The *subacetate of copper* (verdigris) is used to face tea, colour sweetmeats, and give a green appearance to peas and haricot beans. According to M. Kuhlmann, *sulphate of copper* (blue vitriol) has been used by Belgian bakers to promote the rising of bread, and cause it to retain water. One grain to three pounds is the proportion which makes the bread rise best. This salt has also been used on the continent in decolorising syrups made from coarse sugar. The *arsenite of copper* (Scheele's green or emerald green) is used in the "facing" of green tea, and also in colouring confectionery. The *hydrated carbonate* (verditer) is also used to "face" green tea. The *oxichloride* (Brunswick green) is used to colour sugar confectionery. All the preparations of copper are actively poisonous in large doses; when taken in long-continued small doses, they produce symptoms of slow poisoning, which are generally described as characterised by general wasting, and chronic inflammation of the respiratory and digestive organs.

COPPERAS, or Green Vitriol. See *Iron.*

COPPERAS, White. See *Zinc.*

CORIANDER (*Coriandrum sativum),* a plant of the natural order *Umbelliferæ.* The fruit is used to give flavour to gin, instead of the juniper berry. It is not deleterious.

CORN, ROASTED, used in adulteration of coffee and chicory.

DARI, an Egyptian grain, used to adulterate flour.

DARNEL (*Lolium temulentum*), a plant of the natural order *Graminaceæ,* of which it is believed to be the only poisonous species. It is sometimes accidentally mixed with flour, and has produced symptoms of acro-narcotic poisoning.

DEAL, Charred, used in the adulteration of sauce.

DEXTRINE (soluble starch), introduced into milk to increase its density, give a creamy appearance, or remove the bluish tint.

DUTCH PINK, used in the "facing" of green tea.

EARTH NUTS (*Bunium bulbocastanum*), a plant of the natural order *Umbelliferæ.* The oil is used as an adulterant of, or substitute for, olive oil.

EGGS, Whites and Yolks of, are introduced into milk to increase its density, give a creamy appearance, or remove the bluish tint. The adulteration is simply fraudulent.

ELM (*Ulmus campestris*), a tree of the natural order *Ulmaceæ.* The leaves are dried, broken into small pieces, and usually mixed with a paste of gum and starch, then reduced to powder, and used in adulterating tea. It is not deleterious.

EMERALD GREEN (*Arsenite of Copper*), used to colour sugar confectionery. See *Copper.*

ERGOT, a diseased grainshell produced in rye by a peculiar fungus, the *ergotætia abortefaciens.* The use of bread made from ergoted rye has produced, especially in different parts of the continent, fatal poisoning, commonly attended with mortification of the limbs.

FAT (common), frequently used in the form of rancid lard, suet, and tallow, in adulterating cocoa and chocolate. It is best detected by the disagreeable taste and smell. Suet of veal is used to adulterate butter. Adulteration with fat may be suspected, especially if the proportion of concrete fat obtained by boiling half an ounce of cocoa with ten ounces of water exceed from forty to sixty-five grains. The globules of animal oil and fat are large, flat, and disc-like; while those of cocoa-oil are firm, shot-like, nearly globular, and very small. The fat of birds is also used as an adulterant of, or substitute for olive oil. The adulteration is simply fraudulent.

FECULA, the residue obtained from the maceration of plants in water. It may consist of the green or colouring matter of plants—chlorophyll; or of starch—amylaceous fecula. Grey fecula, with pepper husks, powdered hempseed, and beechnuts are mixed in France to form a spurious pepper. Grey fecula is also used for the same purpose mixed with potato blossoms, rape or linseed, and haricot beans. Fecula has nutritive properties.

FELSPAR (*Silicate of Potash and Alumina*), is said to be used by the Chinese in the adulteration of tea. It is not injurious in small proportions.

FLINT (*silica*, combined with *iron*, and occasionally with *alumina*). According to Mitchell, it is employed, powdered, as an adulterant of flour, which naturally contains a small amount of siliceous matter. In large quantities flint would act injuriously, as a mechanical irritant of the intestinal canal.

FLOUR (common wheat flour), is sometimes used in adulteration of sugar, probably to improve the colour of very dark sugar, and to cause the absorption of water, or molasses. It is also employed to adulterate pepper, mustard, chocolate, ginger, arnotto, anchovies, potted bloaters, anchovy paste, lobster and shrimp essence, sugar confectionery, stout and porter, and butter. Baked flour is also used to adulterate honey, milk, and cinnamon. In milk, it is used to increase the density, give a creamy appearance, or remove the bluish tint where water is used. The adulterations are simply fraudulent.

FOOTS, technical name for a coarse brown sugar, used to adulterate stout and porter.

FRENCH FISH, a fish caught off the coasts of Nantes and Nice, is of the same genus as, and used as a substitute for the anchovy. Its commercial value is much less than that of the true anchovy, but its nutritive properties are the same.

FUNGI, Sporules of, microscopic parasitic growths, frequently observed in the less pure kinds of sugar, especially in those which favour the development of acari. They are detected by dissolving a small quantity of brown sugar in water, and examining the sediment by the microscope. It is not ascertained whether these organic forms have any deleterious or other effect on health.

FUSEL OIL (*Hydrated Oxide of Amyle*, produced in the distillation of grain) is used to produce an essence, which has precisely the odour of the finest jargonelle pear. Its effects on the body are not well understood, but in large doses it would possibly be deleterious, producing sedative effects like creasote.

GAMBOGE, the gum of the *Garcinia Cambogia,* natural order *Guttiferæ,* used in powder to colour sugar confectionery. Gamboge, in doses of from six to ten grains, possesses powerful purgative properties. Its continued use in smaller doses is deleterious, and gives rise to intestinal irritation.

GELATINE, an animal jelly prepared chiefly from the bones of oxen, sheep, etc., used in adulterating milk and isinglass. Dr. Hassall found one-third of twenty samples of isinglass to consist of gelatine entirely. Gooseberry jelly is adulterated with, and sometimes wholly formed of, gelatine and pectine, coloured with red beet juice. It is nutritious, and used as a diet for invalids.

GENTIAN, the root of the *Gentiana lutea,* natural order *Gentianaceæ,* used, instead of hops, to render beer bitter. It is simply fraudulent as thus used, being in itself a light and useful tonic.

GINGER, common, the rhizome of the *Zingiber officinale,* natural order *Zingiberaceæ,* used to flavour beer. In large doses it is an irritant; in small, an aromatic.

GLUCOSE (grape sugar), used in the adulteration of honey. See *Sugar.*

GRAIN, Oil of, obtained from the distillation of raw grain, analogous or identical with fusel oil, being amylic alcohol with a salt of amyle and some spirit. It is used to give colour and flavour to whiskey, so as to transform it into a British brandy; it is said also to be used to give the flavour to pine-apple drops. (*Thomson.*)

GRAINS OF PARADISE, Malaguetta pepper, seed of the *Amomum Granum Paradisæ,* natural order *Zingiberaceæ,* a little reddish seed about the size of small shot. The seeds are used in the place of spirit when gin has been diluted, and to give pungency to ales, and to bitter and colour beer. These seeds are not deleterious. They resemble pepper in their action, and are used in the East as a spice for seasoning food.

GRIT, Common earthy, almost constantly present in chicory powder, probably from imperfect cleansing, but also intentionally introduced. It acts as a mechanical irritant on the alimentary canal.

GUIACUM, the wood of the *Guiacum officinale,* natural order *Zygophyllaceæ,* used to render beer bitter (*Chevallier*). The extractive matter derived from guiacum produces sweating and diuresis, when taken in large doses. It is an injurious adulterant.

GUM, the mucilaginous exudation of various plants, is used to cause exhausted tea leaves to assume the twisted form. Gum Arabic is introduced into milk to increase its density, give a creamy appearance, or remove the bluish tint arising from water.

GYPSUM, or Plaster of Paris. See *Lime.*

HAMBURGH POWDER consists of roasted and ground peas, and the like, coloured with Venetian red. It is used for giving colour and increasing substance.

HAY, Common, is used with brown paper to make false cigars.

HAWTHORN (*Cratagus oxyacantha*), natural order *Pomaceæ.* The leaves are dried, broken into small pieces, mixed with a paste of gum and starch, reduced to powder, and used in adulterating tea. The practice is simply fraudulent.

HEMPSEED, the seed of the *Cannabis sativa,* natural order *Urticaceæ.* An emulsion of this seed is used in the adulteration of milk. Hempseed in powder, beech-nuts in powder, grey fecula and husks of pepper, are mixed to form a spurious pepper in France. Hemp or rape cake, with pellitory root, is also used in France to make a factitious pepper. The adulterations are simply fraudulent.

HENBANE (*Conium maculatum),* natural order *Umbelliferæ,* used to adulterate beer. (*Chevallier.)* A rare adulteration; unknown in England. Henbane is a powerful narcotic and intoxicating poison.

HONEY, Common, a product formed by the *Apis mellifica,* or honey-bee, a hymenopterous insect, is used as an adulterant of, or substitute for, olive oil. (*Chevallier.*)

HORSES, Tongues of, killed at the knackers, are probably used for sausages. Perhaps also other parts are used as food. Horse-flesh (sometimes of diseased animals), is used, according to Chevallier, as a substitute for pork and other meats, when made into sausages. The flesh of healthy horses is, in some countries, as Tartary and the northern countries of Europe, a common food. The tendons of horses are supposed to be used for the manufacture of isinglass.

INDIGO, a blue pigment obtained by fermentation from various plants of the genus *Indigofera,* of the Natural Order *Leguminosæ.* The *Indigofera tinctoria* is cultivated in India for supplying the indigo of commerce. Indigo is used in adulterating tea, and in colouring sugar confectionary. In small quantities, varying from a few grains to two or three drachms a day, it has been used medicinally in epilepsy, but without effect. In very large doses it produces vomiting, and in smaller and long continued doses, it causes spasmodic twitching of the muscles, like strychnia; but it is not used in adulteration in quantities sufficiently large to produce these results.

INORGANIC IMPURITIES, a term used to express those impurities which are mechanically suspended or chemically dissolved in fluids, as in water, and which differ from organic impurities by being incombustible.

INSECTS, Remains of, may be accidentally present in honey.

IODINE, an elementary non-metallic substance. It is contained in the salts of bladder-wrack, and is sometimes present in common salt, but possibly not in sufficient quantities to be injurious.

IRON. This simple metallic substance, and its compounds, are common adulterants. *Iron filings* are used to adulterate tea. Dr. R. D. Thomson has examined specimens of tea containing half their weight of iron filings. They may be detected by the magnet. The *red oxide of iron* is frequently present in coffee, cocoa, chocolate, cayenne pepper, anchovies, essence of lobster, and arnotto, being derived from ferruginous earths, such as Venetian red, used in adulteration. It gives a permanency to the colour of the adulterated article. It is likewise used in adulterating Scotch snuff, and may be present accidentally in milk and salt, when iron vessels are used. The *sulphate of iron*, commonly called copperas or green vitriol, is used largely in the adulteration of beer, stout, and porter, to give "head" or froth, and also to give a metallic taste; it is introduced by the publicans. *Ferrocyanide of iron* (Prussian blue) is used in the facing of green tea, and to colour sugar confectionary, and also in the making of Stilton cheese, in order to give the appearance of the blue mould of old cheese. Iron and its preparations in small doses are useful as medicines; the metal enters in fact into the composition of the blood corpuscle, and its use is called for in many cases of debility and anæmia. In large doses, however, iron is a poison. Iron filings act as a mechanical irritant on the intestinal canal; and many of the preparations, in over doses, produce violent effects, as vomiting, purging, burning pain in the stomach, and spasm. An ounce of the sulphate of iron, according to Pereira, produced these effects in a woman who took it for a medicinal purpose; she recovered. The use of iron in small and long continued doses may prove hurtful to persons of a full habit.

ISINGLASS, a gelatinous substance obtained from the air-bladder of some cartilaginous fishes, particularly of the genus *Acipenser*. It is said to be introduced into milk to increase its density, give a creamy appearance, and remove the bluish tint caused by water. This adulteration is doubtful.

JAPAN EARTH. See *Catechu*.

LARD (*Axungia*), the strained and solidified fat of the hog, is used in the adulteration of chocolate, cocoa, and butter. The adulteration is simply fraudulent. See *Fat*.

LEAD. Various preparations of the elementary metallic substance, lead, are used as adulterants. *Minium*, or red lead (a mixed oxide), is used in the adulteration of chocolate, cayenne pepper, snuff, curry powder, cayenne, sauces, pickles, sugar confectionery, and arnotto, principally as a colouring ingredient, and in sufficient quantities to produce the specific effects of lead. Normandy refers to one case of red lead in cheese, resulting from the use of adulterated arnotto. The *carbonate of lead*, or white lead, is stated by Chevallier to be sometimes present in biscuits of a peculiar kind, manufactured at Rheims; being derived from carbonate of ammonia used in their preparation. It is used also to colour sugar confectionery, to increase the weight of butter, and to clarify cider. It is sometimes accidentally present in sugar and beer, from the use of leaden vessels. The *chromate*, or chrome yellow, is used to adulterate snuff, in quantities sufficient to give rise to the peculiar effects of lead as a poison. It is also used to colour sugar confectionery, and to "face" green tea. *Acetate of lead* (sugar of lead), is added to butter to increase the weight. Lead being easily acted upon by vegetable acids, and by water containing carbonic acid gas, deleterious compounds of it not unfrequently result from the use of leaden vessels. Water is thus sometimes impregnated with carbonate of lead, and milk, rum, wine, and cider, may all be charged with salts of lead, from being made or retained in leaden vessels. The dry belly-ache of the West Indies is said to be owing to the impregnation of rum with a salt of lead. Malic acid, the acid present in cider, acts most potently as a solvent of lead. The *oxides of lead* have also been used intentionally to remove the acidity of unsound wines, and to communicate a peculiar reddish-yellow colour to cheese, giving it a "decayed" or mottled appearance; while the acetate is used to clarify spirituous liquors and preserves. All the preparations of lead used in adulteration are poisonous. Given in large doses, as twenty to sixty grains of the acetate, they produce symptoms of irritation of the intestinal canal, and give rise to vomiting, purging, and colic. In long continued doses, however small, they may occasion lead palsy, a result which has occurred from the use of adulterated food. Lead is therefore a most dangerous adulterant.

LENTIL, a plant of the leguminous class, natural order *Leguminosæ*. The seeds and husks powdered, according to Hassall, constitute principally Warton's Ervalenta, Du Barry's Revalenta Arabica, and Edwards Brothers' Arabian Revalenta foods. It is combined with common wheat flour to form Palmer's "Vitaroborant." Lentil possesses slight nutritive powers, but is much inferior to common wheat flour.

LICHENS, cryptogamous plants, genus *Algæ*, natural order *Lichenaceæ*, called commonly rock moss, Iceland moss, and tree-moss. They are used to render beer bitter (*Chevallier.*) The adulteration is simply fraudulent. The lichens have nutritive properties.

LIE TEA, consisting of tea dust, gum, and sand, formed into little masses, and "faced", is manufactured by the Chinese expressly for the adulteration of tea. It may be detected by pouring boiling water on it, when it at once falls to pieces.

LIME (oxide of the metal *Calcium*), is used in the preparation both of brown and loaf sugar, in which it may frequently be detected, notwithstanding careful filtration. Dr. R. D. Thomson has found, in finely crystallised sugar, by no means sweet, from $\frac{1}{2}$ to 1 per cent. He says 10 per cent. will render sugar perfectly bitter. It may be accidentally present in cider. The *carbonate of lime*, or common chalk, is used to adulterate bread, wheat flour, chocolate, cream of tartar, arnotto, snuff, potato-starch, and butter; it is used in the "facing" of green tea. It is also used, though not so frequently as imagined, in the adulteration of milk. It may be accidentally present in cider. It may be detected by means of hydrochloric acid, which forms chloride of calcium, the carbonic acid escaping with effervescence. When oxalate of ammonia is added, an insoluble oxalate of lime is formed. The *sulphate of lime*, plaster of Paris, gypsum, or terra alba, is used in the adulteration of wheat-flour, salt, mustard, chocolate, and potato starch. It is added to flour to increase weight, and has been found by Thomson in proportions from 0·96 to 27·82 per cent. It may be detected by igniting the flour, the ash of which should not be more than 1½ per cent. of the weight of flour used. Sulphate of lime is also used in "facing" green tea, and in sweetmeats and comfits. *Hydrated sulphate of lime* is used in the manufacture of flour, bread, and sugar confectionery. The *phosphate*, in the form of bone-dust, was formerly used in the adulteration of flour. The *oxalate* is an adulterant of Lazenby's Harvey's fish sauce. (*Hassall.*) *Caustic, or quick-lime,* is an adulterant of lard, especially English keg-lard, Irish keg-lard being rarely adulterated. Adulterations of food with lime are simply fraudulent. Introduced in small quantities into the body, lime may even be useful, since it affords the elements of bone. Liebig has lately recommended the use of lime-water in the formation of bread, in the proportion of nineteen parts by weight of flour to five of lime-water, to neutralise the deterioration which the gluten of flour undergoes by keeping, and to supply lime itself to the body.

LIME TREE (*Tilia Europœa,* natural order *Tiliaceœ).* The flowers are used to render beer bitter, in place of hops. (*Chevallier.*) The adulteration is simply fraudulent.

LINSEED, the seed of the flax, *Linum usitatissimum,* natural order *Linaceœ.* Linseed meal and cake are used in the adulteration of pepper and mustard. Linseed meal is also used as an adulterant of rye meal. Linseed or rape seed, grey fecula, potato blossoms, and haricot beans are powdered and mixed to form spurious pepper in France. The adulteration is simply fraudulent.

LIQUORICE (*Glycyrrhiza glabra,* natural order *Leguminosœ).* The juice is used to bitter and colour beer, and is introduced into milk to increase density, give a creamy appearance, or remove the bluish tint, caused by water. Its use is simply fraudulent.

LIVERS of HORSES and BULLOCKS, BAKED, are said to be used in the manufacture of cheap coffee. Fraudulent merely.

LOGWOOD (*Hœmatoxylon Campechianum),* a tree of the natural order *Leguminosœ.* The dust of the wood is used in adulterating chicory and tea. Logwood is an astringent: its continued use is deleterious.

MAGNESIA (Oxide of the metal *Magnesium*). The *carbonate* has been used in the manufacture of bread, in the proportion of from twenty to forty grains to the pound of flour, to improve the colour, and favour the retention of water. The continued use of magnesia is said to promote the formation of stone in the bladder, and of concretions of magnesia in the intestines. Ordinarily it is a simple purgative. The *silicate* (soap-stone or steatite) is used to "glaze" tea. It is detected by its silvery lustre and iridescent appearance under the microscope.

MAIZE, or Indian corn, the *Zea mays*, a graminaceous herb, used in the adulteration of wheat-flour, chocolate, and mustard. Maize is considered to excite, or keep up a tendency to diarrhœa in those unaccustomed to its use. With others, it is nutritious.

MANGEL WURZEL, root of the *Beta hybrida*, natural order *Chenopodiaceæ*, said to be used in the adulteration of coffee and chicory. The adulteration is fraudulent simply. The root is nutritious.

MARIGOLD (a herb of the genus *Chrysanthemum*, natural order *Compositæ*). The flowers are used to colour butter. Tincture of marigold flowers is introduced into milk to increase its density, give a creamy appearance, or remove the bluish tint caused by water. Both adulterations, though not injurious, are deceptive as to quality.

MARSH TREFOIL (*Menyanthes trifoliata*, natural order *Gentinaceæ*), the common buckbean. The leaves are used for colouring and bittering beer. (*Chevallier.*) See *Buckbean*.

MERCURY. The *sulphuret of mercury*, called also vermillion or cinnabar, is used in the adulteration of chocolate, Cayenne pepper, and sugar confectionery, for the purpose of giving colour. All preparations of mercury are most deleterious. In large quantities, they produce intestinal irritation. In smaller ones, and long continued, they accumulate in the system, and produce the well known symptoms of salivation, great modifications in the blood, and degeneration of the tissues.

MICA (*Silicate of Alumina* and *Tersilicate of Potash*), used, it is said, by the Chinese, in the "facing" of tea. It is detected by its silvery lustre and iridescent appearance under the microscope. The effects on the body are not known.

MILK, solidified by heat, is used to adulterate butter. The mixture is simply fraudulent.

MILLET SEED, seed of the *Milium effusum*, natural order *Graminaceæ*, used in the adulteration of flour. The adulteration is simply fraudulent. The seeds are nutritious, and are used as food by the natives of East India and Arabia.

MINERAL GREEN (*Hydrated Subcarbonate of Copper*), used in the "facing" of green tea. See *Copper*.

MUSTARD SEED, seed of the *Sinapis nigra*, natural order *Cruciferæ*, is used in adulteration of pepper, Cayenne pepper, and ginger.

Mustard husks, white and red, are also used to adulterate pepper. Tibbald, a grocer, at Chelmsford, (*Times*, May 10th, 1854) was fined £50 for a cask of pepper of 100 lbs. bulk, but containing only 2 lbs. of pepper, the rest being husks of mustard, chilis, and rice. Mustard, with pepper-dust, or allspice, is rolled up with rapeseed and rye-starch to form spurious pepper-corns, under the name of Lyons pepper. (*Chevallier.*)

NUT OIL, the oil of the *Juglans regia*, natural order *Juglandaceæ*, used as an adulterant of, or substitute for, olive oil. The oil is slightly purgative. The adulteration is simply fraudulent.

NUX VOMICA the plant yielding *Strychnia*, natural order *Apocynaceæ*. See *Strychnia*.

OAK (*Quercus pedunculata*), a tree of the natural order *Cupuliferæ*. The leaves are dried, broken into small pieces, and usually mixed with a paste of gum and starch, then reduced to powder, and used in adulterating tea. The tan of the bark is sometimes used to adulterate coffee. The use of the oak is not deleterious.

OAT (*Avena sativa*), a plant of the natural order *Graminaceæ*. The flower is used in the adulteration of wheat flour. The adulteration is simply fraudulent.

OCHRE, an earth containing peroxide of iron. There are several varieties. *Red* ochre is used in the adulteration of coffee, chocolate, cocoa, Cayenne pepper, snuff, chicory, and arnotto. It is used to give colour and weight. *Yellow* ochre is used in the adulteration of chocolate, snuff, and mustard. Its use is probably not deleterious.

OIL-CAKE, the residue of linseed, from which oil has been extracted, was formerly used in the manufacture of spurious pepper berries, by being mixed with common clay and Cayenne pepper, pressed through a sieve, and rolled in casks. The substitution was simply fraudulent.

OIL OF BITTER ALMONDS, oil of the *Amygdala amara*, natural order *Rosaceæ*, sometimes mixed with spirit, and called essence of bitter almonds. The oil is very volatile, and, as commonly met with, contains prussic acid; Dr. D. Maclagan, however, states that carefully prepared oil of bitter almonds is free from this poisonous substance. From one to two drachms are sufficient to destroy life. It is used extensively for flavouring confectionery. Mr. Streeter has related a case in which it was administered in rice pudding, and nearly destroyed the life of a child. It is a useless and dangerous substance to mix with food.

ORANGE, the fruit of the *Citrus aurantium*, a tree of the natural order *Aurantiaceæ*. The dried peel is used for "gin-flavouring", instead of the juniper berry. It is not deleterious.

ORGANIC IMPURITIES, a general term used to express impurities, in water, sugar, or other articles, derived from organic

(animal or vegetable) matter; they are principally distinguished by being destructible by heat.

ORPIMENT or king's yellow. See *Arsenic, Sulphuret of.*

ORRIS ROOT, the rhizome of the *Iris Florentina*, a liliaceous plant. The powder is used to adulterate snuff and bread. It is an acrid substance, and is liable to produce irritation of the alimentary canal.

PADDY HUSKS (husks of rice), used in the adulteration of tea.

PAPER, BROWN, is mixed with hay and made up and used as cigars. This adulteration is harmless.

PARSNIP (*Pastinaca sativa*), an umbelliferous plant. The root is used in the adulteration of chicory and coffee. It may be detected with care by the presence of starch-granules. It is not deleterious.

PEA, the seed of the *Pisum sativum*, a leguminous plant. The flour is used in the adulteration of coffee, pepper, wheat flour, and sago. In flour it may be detected by the pea odour given off when a little boiling water is poured on the flour to be examined. The adulteration is simply fraudulent.

PELLITORY (*Anacyclus pyrethrum*), a plant of the natural order *Compositæ*. The root is used to bitter and colour beer; with rape or hemp-cake, it is used in France to make a factitious pepper. It has irritant properties, and is injurious.

PEPPER, the dried berry of the *Piper nigrum*, a plant of the natural order *Piperaceæ*. The powder is used to adulterate mustard. The husks of pepper, when ground, are sometimes used as black pepper. The particles are generally much larger than those of the central portion of the pepper berry. They are used in France, mixed with grey fecula, powdered hempseed and beechnuts, to form spurious pepper. P.D., or pepper dust, consists of sweepings of the floors of spice warehouses, or of the siftings of the whole pepper berry. It is mixed with ground pepper. Pepper dust, mustard, or allspice, rolled up with rye-starch and rapeseed, is used in France for spurious peppercorns, under the name of Lyons pepper. The adulteration is simply fraudulent.

PIPE CLAY (*Silicate of Alumina with Potash*), is used to adulterate potato starch and bread. *(Mitchell.)* In bread it may be detected by burning a given weight; the ash should not be more than 1½ per cent.

PLANE (*Platanus*), a tree of the natural order *Platanaceæ*. The leaves are dried, broken into small pieces, and usually mixed with a paste of gum and starch, then reduced to powder, and used in adulterating tea. The adulteration is simply fraudulent.

POPLAR (*Populus nigra*), a tree of the natural order *Salicaceæ*. The leaves are dried, broken into small pieces, and usually mixed with a paste of gum and starch, then reduced to powder, and used in adulterating tea. The adulteration is simply fraudulent.

POPPY (*Papaver somniferum*), a plant of the natural order *Papaveraceæ*. The heads or capsules are introduced into beer to impart to it bitterness and narcotic properties. The oil of poppies is used as an adulterant of, or substitute for, olive oil.

POTASH (an alkali, the oxide of the metal *Potassium*). The *bicarbonate* is used to adulterate lard (especially English lard), snuff, and bread. *Sulphate of potash* is used to adulterate cream of tartar; and *nitrate of potash* (saltpetre) is sometimes introduced into tobacco. The *chromate of potash* (chrome yellow) and *bichromate* (yellow chromate) are used in the facing of green tea, and to colour confectionery. The chromates of potash are highly poisonous: the other salts of this alkali, above mentioned, are not deleterious.

POTATO, the tuber of the *Solanum tuberosum*, a solanaceous plant. The starch or flour is used in the adulteration of coffee, sugar, sago, arrow-root, wheat flour (much so in France), of chocolate, ginger, cinnamon, curry-powder, lard (especially English keg-lard, Irish being rarely adulterated), and sugar confectionery. Boiled and mashed potatoes are often used in the adulteration of bread. This adulteration was much more frequent when the cost of flour was greater. The great objection to this practice is that potatoes are much less nutritious than corn. The ordinary run of bread made with potatoes is at least 20 per cent. less nourishing than that made with good wheaten flour. Potatoes are used also to adulterate butter and cheese. The blossoms of the potato, mixed with green fecula, rape or linseed, and haricot beans, are powdered and mixed to form spurious pepper in France.

PRUSSIAN BLUE (*Ferrocyanide of Iron*). See *Iron*.

QUASSIA (*Picræna excelsa*), a tree of the natural order *Simarubaceæ*. The wood is substituted for hops. The adulteration is simply fraudulent. Quassia is intensely bitter, and forms, medicinally, a light useful tonic.

RADISH. (*Raphanus sativus*, natural order *Cruciferæ.*) The seed is said, but on doubtful proof, to be sometimes used in adulterating mustard. The adulteration is simply fraudulent.

RAPE. (*Brassica napus*, natural order *Cruciferæ*). The seed is sometimes used to adulterate mustard. Rolled up with rye-starch and pepper-dust, mustard, or allspice, it is used in France to form spurious pepper-corns, under the name of Lyons pepper. Rape-seed or linseed, grey fecula, potato blossoms, and haricot beans, are powdered (also in France) and mixed to form a spurious pepper. Rape-seed cake, the residue of the seeds after the expression of the oil, is used to adulterate pepper, and in France, with pellitory root, to make a spurious pepper. The adulteration is simply fraudulent. Rape oil is used as an adulterant of, or substitute for olive oil.

RICE, seed of the *Oryza sativa*, natural order *Graminaceæ*. The flour is used in the adulteration of wheat flour, bread, curry powder, pepper, Cayenne pepper, and ginger. It is also introduced into milk to increase its density, give a creamy appearance, or remove the bluish tint caused by water. Its use is simply fraudulent.

ROOTS, of various plants roasted, are used to adulterate coffee.

ROSE PINK, (colouring matter of logwood, with carbonate of lime), is used to give colour and bloom to the surface of black tea, fabricated from exhausted tea leaves. It is not, however, frequently used, and is not specially injurious to health.

RYE (*Secale cereale,* natural order *Graminaceæ*). Rye flour is used to adulterate wheat flour, in which it may be detected by its specific bitterish taste. Rolled up with rape seed, etc., it forms Lyons pepper. (See *Rape.*) These adulterations are simply fraudulent. Rye flour is a very nutritious substance, when made from the healthy grain.

SAFFRON, the stigmata of the *Crocus sativus,* saffron crocus, natural order *Iridaceæ,* is used to colour butter : though not hurtful, it deceives as to quality. It was once thought to be a powerful medicine, but is now considered inert. When long taken it stains the bones yellow. The common saffron-cake, so called in the shops, is often not saffron at all, but is made up of the tincture of safflower and gum-water rolled into a paste.

SAGO, derived from the *Sagus Rumphii,* natural order *Palmaceæ.* Used in the adulteration of arrowroot, pepper, chocolate, ginger, and cinnamon. The adulteration is fraudulent simply.

SALT, Common (*Chloride of Sodium*), is used to adulterate Cayenne pepper, curry powder, lard (especially English keg-lard), stout, porter, beer, arnotto, and milk.

SALTPETRE *(Nitrate of Potash).* See *Potash.*

SALTS, Earthy, as lime salts, may be present in beer from the water used in its manufacture.

SAND, the fine particles of silicious stones ; used in the adulteration of sugar and honey. White sand has been used to adulterate salt. It is deleterious, acting as a mechanical irritant on the alimentary canal.

SAWDUST of Mahogany, and other woods, used in the adulteration of coffee, chicory, and linseed meal. The adulteration is fraudulent; in large quantities, the substance is possibly a mechanical irritant of the intestinal canal.

SCHEELE'S GREEN (*Arsenite of Copper*), used to colour sugar confectionery. See *Copper.*

SESAMUM, Oil of, derived from the seeds of the *Sesamum orientale,* natural order *Pedaliaceæ,* called also Gingilie oil, is used as an adulterant of, or substitute for, olive oil, and oil of almonds. The adulteration is simply fraudulent.

SICILIAN FISH (a fish, closely allied to the true Gorgona anchovy, but smaller, perhaps of the same genus), substituted for anchovies. The substitution is simply fraudulent.

SIENNA, a brown ferruginous earth, used to colour sugar confectionery. In small quantities it is not injurious.

SLOE (*Prunus spinosa*), natural order *Rosaceæ.* The leaves are used as tea *(Mitchell)*, and as an ingredient of "La Veno Beno". The adulteration is simply fraudulent.

SOAP, Common, (a combination of a fatty substance with an alkali, as soda), used probably to adulterate arnotto. The adulteration is simply fraudulent.

SOAP STONE, or Steatite (*Silicate of Magnesia*). See *Magnesia.*

SODA (oxide of the metal *Sodium*). The *sulphate* (Glauber's salt) is used to adulterate carbonate of soda and common salt. The *bicarbonate* is used to adulterate lard, especially English keg-lard, Irish keg-lard being but rarely adulterated. It is added to flour of an indifferent character, to correct or prevent acidity arising from chemical changes. Sulphate of soda is a laxative, and exists in many mineral waters, as in those of Cheltenham. Bicarbonate of soda, commonly sold as the carbonate in the shops, is diuretic and antacid. As adulterants of food the salts of soda are prejudicial in most cases, though useful medicinally, when properly used.

STARCH, a vegetable product contained as an alimentary principle in many vegetables, and extracted largely from wheat and potatoes. It is used in the adulteration of chocolate, honey, potted bloaters, milk, sugar confectionery, and cocoa. It is also used by the Chinese to cause exhausted tea-leaves to assume the twisted form. In milk, its object is to increase its density, give a creamy appearance, or remove the bluish tint caused by water. The adulteration is fraudulent simply, starch being highly nutritious.

STEEL, in powder, is used to adulterate cocoa. See *Iron.*

STONE, Fragments of, or Grit, almost constantly present in brown sugar. They may be derived from the imperfectly washed cane, from the lime used in clarifying the juice, or from the clay used in the process of claying. In large quantities they would act as mechanical irritants on the intestinal canal.

STRYCHNINE, an alkaloid derived from the *Strychnos nux vomica,* natural order *Apocynaceæ,* supposed by some to be used instead of hops, for rendering beer, ale, and especially the bitter ales, bitter. This adulteration has, however, never been proved, though Normandy has obtained evidences sufficient to make it exceedingly probable. Strychnia is one of the most active poisons, producing symptoms resembling lock-jaw. A single grain, or even less, may prove a poisonous dose. In small doses, such as the eighth or sixteenth of a grain, it is considered to be a tonic, and is used in cases of paralysis.

SUET, the loin fat of animals. Suet of veal is used to adulterate butter. See *Fat.*

SUGAR, obtained principally from the *Saccharum officinale*, natural order *Graminaceæ*, but also from various other plants, such as maple and beet. Sugar is used to adulterate chocolate, cocoa, milk, tobacco, and gin, and a spurious cider is also made from it, in connexion with vinegar and canella. Burnt sugar or *Caramel* is largely prepared for the adulteration of chicory and coffee, and to impart colour and bitterness to the decoction of inferior articles sold as coffee. It is also used in the adulteration of vinegar. *Grape sugar* or *glucose*, a sugar existing in, and extracted from grapes and honey, and also procured in large quantities from the decomposition of cane sugar itself, and from the action of dilute mineral acids on starch, or woody fibre, is frequently met with in large quantities in brown cane sugar. Grape sugar is sometimes used to adulterate gooseberry syrup, which is much used in France. Sugar cane in fragments is frequently met with in impure sugar. Sugar is introduced into milk to increase its density, give a creamy appearance, and remove the bluish tint arising from water. "Foots", a coarse form of sugar, is used to adulterate ale and porter, to deepen the colour, and give a sweetish taste. These adulterations are rather fraudulent than deleterious.

SULPHURIC ACID, or Oil of Vitriol, a mineral acid, is used in the adulteration of vinegar, pickles, stout and porter, and to "bead" gin. In small doses, one or two drops, largely diluted with water, this acid is an astringent and tonic. In large doses, varying from five to sixty drops, it produces very deleterious effects, and sixty drops have been known to prove fatal. It acts as a corrosive poison, destroying the surface of the stomach. Its long continued use cannot fail to prove injurious in the majority of cases.

TALC (*Silicate of Alumina and Tersilicate of Potash*) is said to be used in the adulteration of tea by the Chinese. See *Mica.* Venetian talc (*Silicate of Magnesia*) is used to glaze tea. The adulteration is simply fraudulent.

TAPIOCA, the starch of the root of the *Janipha manihot*, an euphorbiaceous plant. It is used in the adulteration of arrow-root and cocoa. It is not deleterious.

TARTARIC ACID, a vegetable acid, obtained principally from cream of tartar, and existing in many vegetables. It is sometimes used to adulterate gooseberry syrup, which is much used in France. (*Chevallier.*) The adulteration is simply fraudulent. Tartaric acid in moderate quantities is useful as an article of diet, preventing scorbutic disorders.

TEA LEAVES, exhausted, are dried and often employed in the manufacture of spurious tea. The fraud is readily detected by observing the greatly diminished quantity of tannin, and the increased amount of lignin and of gum.

TERRA ALBA. See *Lime.*

TRAGACANTH, also called gum dragon (gum of the *Astragalus verus*, or milk vetch, natural order *Leguminosæ*,) sometimes used in the adulteration of milk; also used in the adulteration of honey. Its use is simply fraudulent.

TREACLE, or molasses, the uncrystallisable substance produced in the manufacture of sugar. It forms with salt, the basis of, if it is not substituted for Indian soy. It is also used to adulterate stout and porter. The adulteration is principally fraudulent. In large quantities it might give rise to indigestion and acidity.

TURMERIC, the powdered tuber of the *Curcuma longa*, a plant of the natural order *Zingiberaceæ*. It is used largely in the adulteration of mustard, Cayenne pepper, curry powder; also of tea, milk, sauce, ginger, and arnotto. The adulteration is simply fraudulent.

TURNIP, the *Brassica rapa*, natural order *Cruciferæ*, is used largely to adulterate orange marmalade. Turnips are formed into rolls, and roasted, to form spurious roasted onions in France. (*Chevallier.*) Turnips are also given to milch cows as food; the colouring matter, being imparted to the milk, gives it a more creamy and richer appearance.

ULTRAMARINE, German or Artificial (*Sulphuret of Sodium* and *Aluminum*), used to colour sugar confectionery. The effects are not known, but in large doses it would probably act as an irritant and a narcotic poison.

UMBER, a brown pigment or earth, consisting of oxides of iron, manganese, water, and silicate of alumina, is obtained from Ombria in Italy, and from Cologne. It is used to adulterate snuff, and to colour sugar confectionery. It is not specially injurious in small quantities; in large quantities it acts as an irritant.

VANDYKE BROWN, a brown ferruginous earth, used to colour sugar confectionery. It is not injurious.

VENETIAN RED. See *Iron*.

VENO BENO consists of sumach leaves (leaves of the *Rhus toxicodendron*, or trailing poison oak, natural order *Terebinthaceæ*) and catechu (gum of the acacia catechu, natural order *Leguminosæ*). (*Phillips.*) Catechu possesses powerful astringent properties, and is extremely apt to produce constipation; and the leaves of the sumach produce, in doses of from half a grain to one grain, diuresis, and increased action of the skin; in large doses, vomiting and stupefaction. Veno beno is a dangerous substitute for tea.

VERDIGRIS (*Subacetate of Copper*), used in the "facing" of green tea, and in colouring sweetmeats. It has also been used to give a green colour to peas and to haricot beans. See *Copper*.

VERDITER (*Hydrated Carbonate of Copper*). See *Copper*.

VERMILLION. See *Mercury.*

VIBRIONES (infusorial animalcules). Chevallier states, on the authority of M. Fuchs, that a peculiar blue or yellow colour sometimes observed in milk is due to the presence of these animalcules. The effects are not known.

VINEGAR (impure acetic acid), and sugar, aromatised with canella, are sometimes used to make a spurious cider. See *Sugar.*

VITRIOL, Blue. See *Copper.*

VITRIOL, Green. See *Iron.*

VITRIOL, Oil of. See *Sulphuric acid.*

VITRIOL, White. See *Zinc.*

WATER, used in the adulteration of milk, vinegar, lard (especially English keg-lard, Irish keg-lard being rarely adulterated), butter, tobacco, snuff, stout and porter, gin, rum, and ale. The adulteration is simply fraudulent.

WAX, a secretion formed by the *Apis mellifica,* or honey-bee. It may be present in honey, from imperfect purification.

WHEAT (*Triticum commune*), a graminaceous plant. The hulls, or husks of the grain, are used to adulterate flour.

WILLOW (*Salix caprea*), a tree of the natural order *Salicaceæ.* The leaves are dried, broken into small pieces, and usually mixed with a paste of gum and starch, then powdered, and used in adulterating tea. The adulteration is simply fraudulent.

WOODY FIBRE is present, probably accidentally, in brown sugar, derived from the cask in which it is contained. Microscopic fragments of woody fibre are almost constantly present in lump sugar, frequently in great abundance. They may be designedly introduced to promote crystallization. *(Hassall.)*

ZINC, a metallic elementary substance. Salts of this metal are sometimes accidentally present in milk, from keeping it in zinc pans. The *sulphate of zinc* (white vitriol), is used to give "bead" to gin. Mitchell says bread is adulterated with it. In small doses, one or two grains, sulphate of zinc is a metallic tonic. In larger doses, twenty grains, it produces vomiting; and, in still larger doses, it produces vomiting, purging, and all the symptoms of an irritant poison. In small and long continued doses, it would prove deleterious, as a general rule. It may be classed as a deleterious adulterant.

